# Active Thermal Extraction and Temperature Sensing of Near-field Thermal Radiation

**DOI:** 10.1038/srep32744

**Published:** 2016-09-06

**Authors:** D. Ding, T. Kim, A. J. Minnich

**Affiliations:** 1 Division of Engineering and Applied Science, California Institute of Technology, Pasadena, California 91125, USA

## Abstract

Recently, we proposed an active thermal extraction (ATX) scheme that enables thermally populated surface phonon polaritons to escape into the far-field. The concept is based on a fluorescence upconversion process that also occurs in laser cooling of solids (LCS). Here, we present a generalized analysis of our scheme using the theoretical framework for LCS. We show that both LCS and ATX can be described with the same mathematical formalism by replacing the electron-phonon coupling parameter in LCS with the electron-photon coupling parameter in ATX. Using this framework, we compare the ideal efficiency and power extracted for the two schemes and examine the parasitic loss mechanisms. This work advances the application of ATX to manipulate near-field thermal radiation for applications such as temperature sensing and active radiative cooling.

Heat transport by electromagnetic radiation is a fundamental process that plays a role in numerous applications such as thermal management in space^1^,^2^, radiative cooling and heating^3^,^4^,^5^,^6^,^7^, and energy conversion^8^. Of particular interest is how electromagnetic radiation can be used to transport heat either in passive or active schemes, and the limits of the heat transport in these schemes. For instance, although the far-field heat flux cannot exceed the blackbody limit, a number of works have experimentally demonstrated that passive near-field radiative heat transfer is enhanced by many orders of magnitude compared to the far-field limit for closely spaced objects with either natural^9^,^10^,^11^,^12^ or engineered resonant surface modes^13^. These effects have been exploited for enhanced near-field radiative cooling but are limited to the near-field^14^,^15^,^16^. In active schemes, laser cooling of solids (LCS) enables active cooling with light by removing thermal energy in the form of phonons with upconversion fluorescence^17^ but is only possible in certain solids.

Recently, we theoretically proposed active thermal extraction (ATX) scheme that allows near-field electromagnetic surface waves to propagate into the far-field, thereby enhancing the total radiative flux emitted by a solid^18^. The technique operates by exploiting the monochromatic nature of near-field thermal radiation to drive a transition in a laser gain medium that, when coupled with external optical pumping, allows the resonant surface mode to be emitted into the far-field.

ATX shares many similarities with LCS, particular its ability, in principle, to cool an object below ambient temperature, but is applicable to a wider range of solids than LCS.

LCS was first demonstrated experimentally by Epstein *et al.*^19^ in ytterbium-doped fluorozirconate glass and has since been experimentally demonstrated to cool other rare-earth doped glasses^20^,^21^,^22^,^23^,^24^,^25^,^26^,^27^,^28^,^29^,^30^,^31^,^32^,^33^,^34^,^35^,^36^,^37^ and recently to cool semiconductors^38^ and lead perovskites^39^. At the same time, LCS has been used as a means to measure temperature by observing the wavelengths of emitted light, with applications for temperature sensing at the nanoscale and in biological tissues^40^,^41^,^42^,^43^,^44^,^45^,^46^,^47^.

In this work, we apply the mathematical framework of LCS^34^,^36^ to create a generalized model of ATX. We show that LCS and ATX can be described with the same mathematical formalism by replacing the electron-phonon coupling parameter in LCS with the electron-photon coupling parameter in ATX. We then examine how ATX may be used for applications such as radiative cooling and temperature sensing.

This paper is organized as follows. We first summarize the derivation of the model for LCS. Then, we derive an analogous generalized model for our ATX scheme. We next explain the mathematical equivalence between electron-phonon coupling model in LCS and electron-photon coupling model in ATX, examine the potential of ATX for near-field extraction in terms of efficiency and net power, and discuss how parasitics can affect the performance of ATX. Afterward, we consider how ATX may be used for non-contact temperature sensing. Finally, we end with a summary of the results.

## Theory

### Generalized theory for laser cooling of solids

A two-level model to analyze LCS has been given by Luo *et al.*^21^ and was further developed by Sheik-Bahae and Epstein into a generalized four-level model of LCS^34^. Models that have taken other factors into account such as excited state absorption^36^ or thermodynamics^48^,^49^ will not be discussed in the paper. The goal of this section is to briefly highlight the LCS model in ref. 34 in order to facilitate the derivation in the next section for ATX.

**Figure 1:**
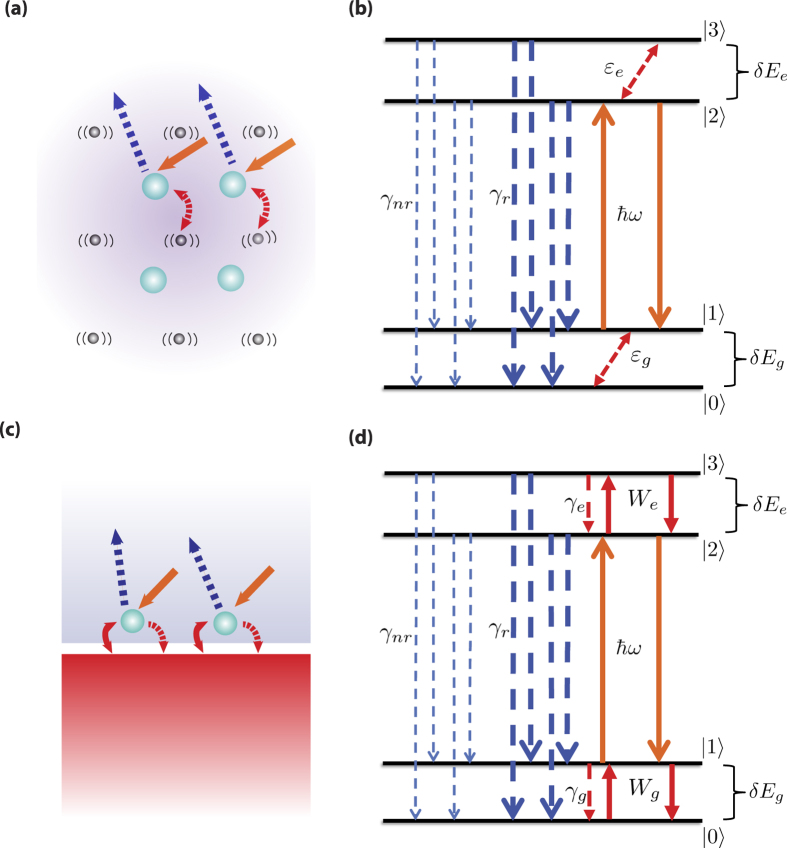
(**a**) Schematic of the concept in laser cooling of solids (LCS). The gain medium consists of rare earth emitters embedded in a host material at a finite temperature. The external pump photons excite the rare-earth emitter, and by absorption of a phonon, carry the energy away as upconverted fluorescence. (**b**) Energy diagram of the four-level system for LCS. A incident pump laser excitation with energy *ħω* is shown by the solid orange arrow. The thick dark blue dashed arrows indicate spontaneous emission transitions with a rate of *γ*_*r*_ and the thin blue dashed arrows indicates the nonradiative decay rates (*γ*_*nr*_). *ε*_*e*,*g*_ is the electron-phonon coupling rate with the subscript “g” for the ground state manifold |0〉 and |1〉 and “e” for the excited state manifold |2〉 and |3〉, respectively. (**c**) Schematic showing the concept of active thermal extraction (ATX). A rare-earth doped gain medium is placed in the near-field of a substrate. The external pump photons excite the rare-earth emitter and result in blue-shifted fluorescence due to coupling to the near-field thermal radiation from the substrate, leading to extraction of thermal energy. (**d**) Energy diagram of the four-level system for ATX. *γ*_*e*,*g*_ is the overall decay rate and *W*_*e*,*g*_ is the absorption and stimulated emission rate for each of the manifold. The subscripts “e” and “g” refer to the same manifolds as (**b**).

The basic principle of LCS is illustrated in Fig. 1(a). The gain medium consists of emitters embedded in a host lattice at finite temperature. The energy of the lattice due to its finite temperature will manifest as phonons or vibrations of the lattice atoms. These vibrations will couple to the emitters through perturbations of the valence electrons, exchanging energy with the emitters. The net result of this interaction is thermal equilibrium of the electron with phonons in the host. When an incident pump is introduced into the gain medium, the valence electron is excited to a higher energy level. It may in turn absorb a phonon and then emit upconverted light, thereby extracting thermal energy from the system.

Figure 1(b) shows the four-level system of Fig. 1(a) for LCS for applications of cooling in refs 34, 36. The ground state manifold consists of two closely spaced levels of |0〉 and |1〉 separated by energy *δE*_*g*_, and the excited manifold consists of |2〉 and |3〉 with an energy separation *δE*_*e*_. The subscript “e” and “g” indicates the excited or ground state manifold, respectively. A incident pump laser excitation with energy *ħω* is on resonance with the |1〉-|2〉 transition. The spontaneous emission transitions are labeled as *γ*_*r*_ and likewise the non-radiative decay rates are labeled *γ*_*nr*_. The electron-phonon interaction rates are given by *ε*_*g*_ and *ε*_*e*_. We assume unity degeneracy for all levels and let the overall decay rate *R* = 2(*γ*_*r*_ + *γ*_*nr*_). The rate equations for the density populations *N*_0_, *N*_1_, *N*_2_, and *N*_3_ are:


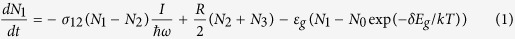



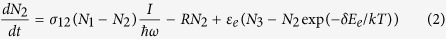



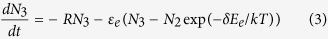



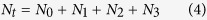


where *σ*_12_ is the absorption cross section of the |1〉-|2〉 transition, *I* is the incident laser intensity, *k* is the Boltzmann constant, *N*_*t*_ is the total emitter density and *T* is the lattice temperature. Evaluating the steady-state solution to Eqs 1, 2, 3, 4, we define the net power density as the difference between absorbed and radiated contributions as


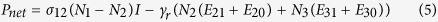


We have ignored a term that represents parasitic absorption of the pump laser in refs 34, 36 for the purpose of illustrating the concept of LCS. The net power density can then be expressed as


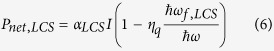


where 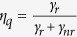 is the internal quantum efficiency of the transition and *ħω*_*f*_ denotes the mean fluorescence energy of the four-level system given by


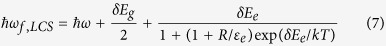


with the ground state resonant absorption *α*_*LCS*_ given by


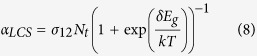


In deriving Eq. 6, we ignore saturation as in refs 34, 36.

The cooling efficiency is defined by *η*_*LCS*_ = −*P*_*net*,*LCS*_/*P*_*abs*,*LCS*_ with *P*_*abs*,*LCS*_ = *α*_*LCS*_*I* and from Eq. 6


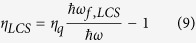


Other than cooling, the upconverted fluorescence that occurs in LCS has also been exploited for temperature sensing^40^,^41^,^42^,^43^,^44^,^45^,^46^,^47^. In this case, the gain medium is in thermal equilibrium with the medium of interest. Again solving Eqs 1, 2, 3, 4, we can obtain the upconverted output power as


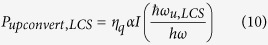


where the up-converted mean photo-luminescence energy *ħω*_*u*_ is now defined as


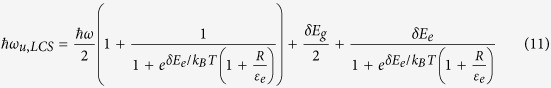


unlike Eq. 7. For temperature sensing, the sensitivity of the emitted fluorescence to variations in temperature *dP*_*upconvert*,*LCS*_/*dT* is the key parameter rather than the net extracted power. Taking ratios of the upconverted intensity with a reference is the widely used method today^40^,^41^,^42^,^43^,^44^,^45^,^46^,^47^ but for simplicity we will focus on the absolute upconverted power for the generalized model here.

### Generalized theory for active thermal extraction

Active thermal extraction (ATX) in Fig. 1(c) employs a laser gain medium containing emitters with discrete energy levels placed in the near-field of a material that supports a resonant surface wave. We assume no physical contact between the gain medium and the substrate so that thermal radiation is the only form of heat transfer between them. Similar to LCS, the emitters here exchange energy and thus are in quasi-thermal equilibrium with the thermal near-field. With external pumping, the near-field energy absorbed by the emitter can combine with the pump to be remitted as blue-shifted light into the far-field^18^.

We model the emitters in our ATX scheme as a four-level system, as shown in Fig. 1(d). An external pump laser is tuned to the |1〉-|2〉 transition. The near-field thermal radiation drives the transition from |0〉-|1〉 and |2〉-|3〉. Two of the four spontaneous emission channels in Fig. 1(d), namely |3〉-|0〉 and |2〉-|0〉, will emit blue-shifted photons in the far-field thereby extracting thermal energy out of the system.

The generalized system of equations for the scheme in Fig. 1(d) can be written as


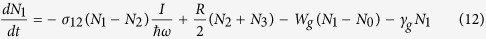



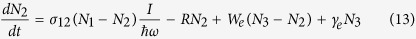



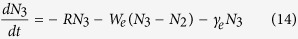



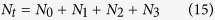


where quantities are defined in the same way as Eqs 1, 2, 3, 4. The ground state manifold (|0〉 and |1〉) and the excited state manifold (|2〉 and |3〉) in Eqs 12, 13, 14, 15 are coupled to near-field thermal radiation. Spontaneous emission rates *γ*_*g*_ and *γ*_*e*_ are associated with the ground and excited state manifold, respectively. Absorption and stimulated emission associated with each manifold are defined as *W*_*g*_ and *W*_*e*_, respectively. Absorption and stimulated emission for each manifold are equal assuming unity degeneracy: *W*_01_ = *W*_10_ = *W*_*g*_ and *W*_23_ = *W*_32_ = *W*_*e*_.

Solving Eqs 12, 13, 14, 15 in steady state and using the same definition of net power as Eq. 5, one can express the net extracted power for ATX in the same form as Eq. 6.


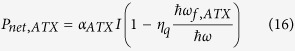



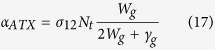


where *α*_*ATX*_ is the ground state absorption for the ATX model. The mean fluorescence energy *ħω*_*f*,*ATX*_ for ATX is given by


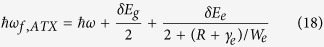


Likewise, the efficiency can be defined in the same way as Eq. 9:


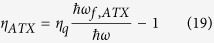


with the mean fluorescence energy defined in Eq. 18 above.

In addition, we can quantify the potential for ATX for temperature sensing applications through the upconverted output power like Eq. 10 in LCS as follows:


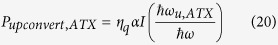


where the corresponding up-converted mean photo-luminescence *ħω*_*u*,*ATX*_ is


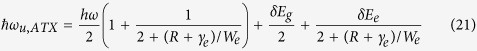


The ability for ATX to sense temperature changes is denoted by its sensitivity to temperature change *dP*_*upconvert*,*ATX*_/*dT* which will be discussed in the subsequent sections.

### Comparision of ATX and LCS

With the theory for a generalized LCS system and a generalized ATX system established in previous two sections, we now explore the relationship between the two schemes. Intuitively, a close correspondence should exist between LCS and ATX because the fluorescence up-conversion process in the two schemes is identical. The key difference between the two schemes is the energy of the extracted particle and the nature of the coupling between the electrons and the emitters. In LCS, phonons with relatively small energies on the order of meV (~10 meV) are extracted and the quasi-thermal equilibrium electron-phonon coupling constants between states |0〉-|1〉 in the ground state and |2〉-|3〉 in the excited state manifold are the relevant parameters. In ATX, the extracted quasiparticles are surface phonon-polaritons with energies on the order of hundreds of meV, and the coupling constants are the radiative spontaneous and stimulated decay rates of the energy levels of the emitters due to the emission of photons.

We now examine the comparison in more detail. If we neglect the excited state manifold and just focus on the ground state manifold |0〉 and |1〉 in Fig. 1(b), we can write a rate equation for the two-level case for LCS as:


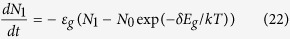


Similarly, isolating the ground state manifold in the ATX case in Fig. 1(d), we have a two-level system |0〉 and |1〉 coupled to thermal radiation with the rate equation for state |1〉 as:


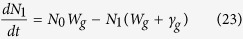


Examining Eqs 22 and 23, we find that they can be made identical with the following substitutions:


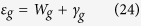



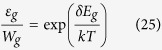


Therefore, the electron-phonon coupling rate *ε*_*g*_ in LCS takes the role of the spontaneous and stimulated rates *γ*_*g*_ and *W*_*g*_ for electron-photon coupling with thermal radiation in ATX.

Examining Eqs 24 and 25, we can relate spontaneous and stimulated rates *γ*_*g*_ and *W*_*g*_ using the Boltzmann factor as:


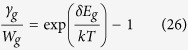


On the other hand, if for ATX we assume that the ground state is in quasi-thermal equilibrium with the thermal radiation such that


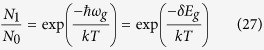


we can also obtain Eq. 26 from substituting Eq. 27 into Eq. 23. Thus, quasi-thermal equilibrium is automatically guaranteed in the mathematical equivalence in Eqs 24 and 25. Also, Eq. 26 is identical to the classical result for a two-level system interacting with thermal radiation^50^. In Einstein’s work^50^, only the far-field form of thermal radiation described by Planck’s law was considered, but the formulation depends only on the photonic density of states and thus is applicable in the near-field as well. Here in ATX, radiative thermal equilibrium is assumed between thermal radiation of the substrate and the emitters of the gain medium.

Although there are many similarities between LCS and ATX, there is one important difference. In LCS, the relevant temperature for the extracted thermal phonons is that of the gain medium itself. In ATX, the relevant temperature is that of thermal radiation emitted from substrate, which may be very different from that of gain medium if, for instance, the medium is maintained at a given temperature by a separate thermal reservoir. This difference in temperature can have important implications, particularly for the strength of non-radiative processes that depend on the temperature of the gain medium, and will be discussed in a later section on parasitic losses.

## Results

### Ideal efficiency and extracted power

We now compare the ideal efficiency and net extraction power that can be achieved with LCS and ATX using the mathematical formalism derived in the previous section, neglecting the influence of parasitic processes. These processes will examined in the next section. To perform this comparison, we need to choose realistic parameters for the gain media for both LCS and ATX. Due to the considerable differences in requirements of the gain media for LCS and ATX, it is not possible to directly compare LCS and ATX based on the same gain medium. For instance, the host material for the gain medium for LCS does not have to be transparent in the mid infrared (MIR) should be transparent in the wavelength range for ATX. This transparency ensures that the near-field thermal radiation can interact directly with the emitters rather than be absorbed by the host material.

First, we estimate the energy *δE*_*g*_ and *δE*_*e*_ for the ground and excited state manifolds assuming that they are approximately equal. For LCS, typical phonon energy of rare earth materials such as doped fluorozirconate glass (ZBLAN:Yb^3+^) is around a few percent of the pump photon energy^19^. Here, we assume a typical value of *δE*_*g*_ ≈ *δE*_*e*_ ~ 0.01*ħω* for LCS.

For ATX, the typical thermal photon energy is higher than the phonon energy and ideally has a value that is close to the energy corresponding to the peak of the blackbody spectrum^18^ so as to maximize its near-field energy density. If we consider the temperature of the substrate to be 300 K and choose a rare-earth emitter with transitions that matches the peak of the blackbody spectrum peaks around 10 *μ*m, the corresponding manifold energy separation *δE*_*g*_ ≈ *δE*_*e*_ ≈ 0.1*ħω* assuming a pump wavelength of 1 *μ*m. Thus, we observe that the energy gaps in ATX are at least a few times larger than the energy gaps of those in LCS due to the larger energies of surface phonon polaritons compared to those of phonons.

To estimate the decay rates for LCS and ATX, we have to examine each coupling mechanism. Having established mathematical equivalence of LCS and ATX, Eq. 7 only requires us to estimate the values of the spontaneous decay rate *R* and the decay rate *ε* for external coupling within the manifold (assuming *ε*_*g*_ = *ε*_*e*_ = *ε*). For LCS, this coupling is provided by electron-phonon interaction. Here, *ε* follows the energy gap law^51^ given by


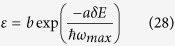


where *a* and *b* are constants and *ħω*_*max*_ is the maximum phonon energy of the host material. Typical values of *a* and *b* are on the order^52^,^53^ of 3.5 and 10^12^, *ω*_*max*_ ≈ *ω*/10 and *δE* ≈ 0.01*ħω*. Using these parameters, we estimate the electron-phonon coupling to be *ε* ∼ 10^11^ which is within the range of values for known host materials^52^. Considering typical *γ*_*r*_ to be on the order^53^ of 100 s^−1^ and assuming a unity quantum efficiency, the overall decay rate *R* = 2*γ*_*r*_ = 200 s^−1^ which is much smaller than *ε*. Thus, the mean fluorescence efficiency in Eq. 7 can be approximated as:


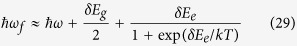


For ATX, the coupling rate *ε* within each manifold is the sum of the spontaneous and stimulated rates (*γ* and *W*) according to Eq. 24. Like ref. 18, we assume that the surface resonance of the substrate in Fig. 1(c) matches the energy separation *δE*_*g*_ ≈ *δE*_*e*_ of each manifold. As a result, the enhanced density of states in the near-field will increase both *γ* and *W* by orders of magnitude^18^. Using Eq. 24, the coupling *ε* within each manifold for our scheme will also be orders of magnitude larger compared to the overall decay rate *R* if we again assume *R* to be around 200 s^−1^. Thus, we can define the mean fluorescence frequency in the same way as was done for LCS in Eq. 29 as:


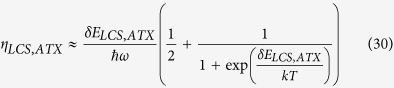


and likewise express net extracted power normalized with respect to incident absorbed power as


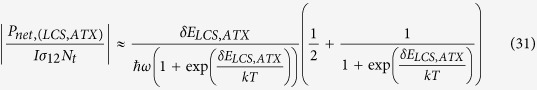


assuming the quantum efficiency *η*_*q*_ = 1.

**Figure 2:**
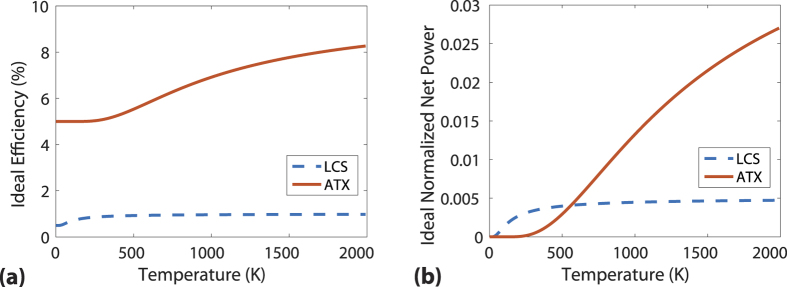
(**a**) Ideal efficiency versus temperature for LCS (dashed line) and ATX (solid line) from Eq. 30. (**b**) normalized extracted ideal net power versus medium temperature of LCS (dashed line) and ATX (solid line) from the absolute value of Eq. 31. ATX has a higher ideal efficiency than LCS but LCS outperforms ATX for extracted power at lower temperatures.

Figure 2(a) shows the comparison of the ideal efficiency, without consideration of parasitics, versus temperature for ATX and LCS using Eq. 30 with pump energy *ħω* = 1.24 eV for both schemes. The overall higher ideal efficiency for ATX is due to higher energy of the extracted phonon polariton compared to that of typical phonons. In the limit of large temperature, the ideal efficiency for both LCS and ATX tends to *δE*/(*ħω*) according to Eq. 30 which is 10% for ATX and 1% for LCS as shown in Fig. 2(a). In the limit of low temperatures, the ideal efficiency tends to *δE*/(2*ħω*) according to Eq. 30. This limit is also obeyed as shown in Fig. 2(a) which is 5% for ATX and 0.5% for LCS.

To compare the ideal net power, we use the form of normalized power with respect to incident absorbed pump power as defined in Eq. 31 and plot the extracted ideal net power |*P*_*net*_/(*Iσ*_12_*N*_*t*_)| as shown in Fig. 2(b). Figure 2(b) shows that at higher temperatures more power can be extracted using our ATX scheme compared to that with the LCS scheme. However, at lower temperatures then LCS extracts more power than does ATX. These results are expected since if when 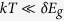 the excited state of the manifold will be depopulated as discussed in ref. 34 and in the section “Generalized theory for laser cooling of solids”. The higher energy gap *δE* in ATX means that this depopulation occurs at higher temperatures compared to the relevant depopulation temperature for LCS.

### Parasitic losses

Thus far, we have neglected non-idealities such as parasitic pump absorption and non-unity quantum efficiency. In reality, these process will degrade the performance of both LCS^34^ and ATX for cooling and temperature sensing applications. We now examine these effects.

The key parasitic losses in LCS are parasitic pump absorption and non-radiative recombination of upconverted photons (manifested by a non-unity quantum efficiency), and both of these processes will occur in ATX as well. We first consider parasitic absorption of the pump. Here, the pump wavelength here is chosen to be 1 *μ*m (1.24 eV) and most host materials such as ZBLAN or YLF^34^ are transparent at this wavelength in LCS. In ATX, the requirement for the host materials to be transparent up to MIR limits host materials to those that are 100% transparent at 1 *μ*m such as calcium fluoride. In ATX, however, there is also the possibility of pump absorption by the substrate in a simple geometry such as in Fig. 1(c). The details of how much pump absorption occurs depends strongly on the material properties and system design. However, it is clear that cooling applications using ATX will require thin substrates that do not absorb light in the visible or near-infrared wavelengths used for the pump.

Next, we consider non-radiative recombination of upconverted photons. These non-radiative channels are represented by *γ*_*nr*_ for the all transitions in Fig. 1(b,d) and are caused by multi-phonon decay processes governed by Eq. 28. Upconverted photons require at least 97% internal quantum efficiency (assuming unity absorption efficiency and fluorescence escape efficiency) in order for any cooling to occur in LCS^34^. Thus, host materials in LCS often have low maximum phonon energy to reduce the probability of multi-phonon processes^53^. In ATX, the mean fluorescence energy is larger than in LCS due to a larger energy gap *δE*, which should result in a reduction in parasitic multi-phonon decay processes.

However, the elevated temperatures required for optimal performance of ATX could lead to a dramatic increase in non-radiative recombination. This challenge may be avoided by recognizing that the temperature of the host medium need not equal that of the thermal radiation emitted by the substrate. In ATX, the substrate determines the thermal photon population, unlike LCS where the physical temperature of the host material of the gain medium that determines the phonon population. Thus for ATX, only emitters in the gain medium are in radiative thermal equilibrium with the substrate. Thus, the host material in ATX can be maintained at a lower temperature compared to that of the substrate by contact with a thermal reservoir. As a result, non-radiative recombination may be significantly smaller than anticipated despite the elevated temperature of the substrate. The temperature of the gain medium primarily affects the operation of ATX by setting the rate of non-radiative decay processes, which in turn affects the efficiency and extracted power.

Overall, parasitic losses should affect LCS and ATX to a similar extent and it is possible that radiative cooling could be achieved with ATX. Nevertheless, specialized experimental design plays a key role in achieving cooling in LCS^19^,^20^,^21^,^22^,^23^,^24^,^25^,^26^,^27^,^28^,^29^,^30^,^31^,^32^,^33^,^34^,^35^,^36^,^37^,^38^,^39^ and similar careful design will be required for achieving cooling using ATX.

## Discussion

With the mathematical formalism in place and the parasitic processes in mind, we now examine the applications of ATX for temperature sensing. The key quantities are the upconverted power reaching the detector and the sensitivity of the upconverted power to variations in temperature. Using the same assumptions in the section “Ideal efficiency and extracted power”, we simplify Eqs 10 and 20 to obtain the upconverted power normalized to absorbed input power as


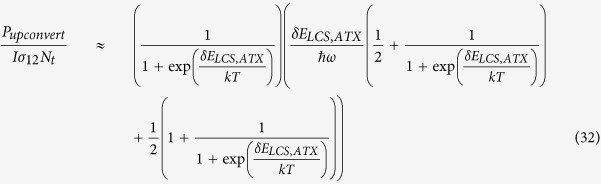


The sensitivity of upconverted power to variations in temperature defined as *dP*_*upconvert*_/*dT* is then


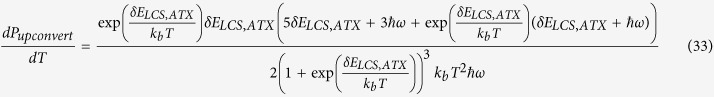


**Figure 3:**
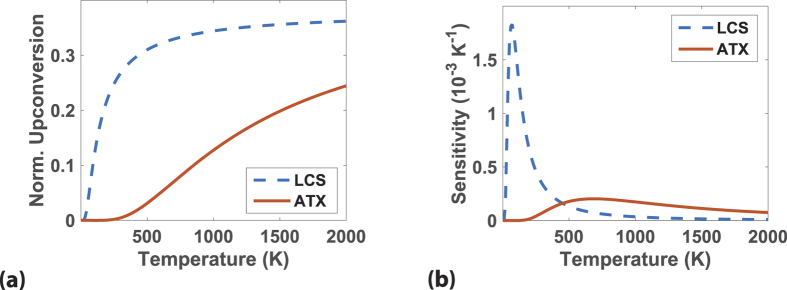
(**a**) Normalized upconverted power versus temperature for LCS (dashed line) and ATX (solid line) from Eq. 32. (**b**) Sensitivity of upconverted fluorescence versus sensing temperature of LCS (dashed line) and ATX (solid line) from the absolute value of Eq. 33. ATX has a higher sensitivity than LCS at higher temperatures but LCS outperforms ATX for extracted power for the temperature range considered.

Figure 3(a) shows the comparison of the normalized upconverted power and radiation temperature for ATX versus LCS using Eq. 32 with pump energy *ħω* = 1.24 eV for both schemes. The higher power output for LCS is due to the smaller energy of the manifold that allows a higher thermal population of the excited state. In Fig. 3(b), the sensitivity of LCS is lower than ATX at higher temperatures although it is much higher below 500 K.

The factors discussed in the section above on parasitic losses applies to temperature sensing in the same way where non-idealities can lead to a decrease in the amount of upconverted signal. Keeping the temperature of the gain medium constant with a separate thermal reservoir is thus advantageous in reducing losses at elevated temperatures. Overall, the comparison here shows that LCS is better for temperature sensing for the temperature range considered as 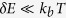. If contact between the fluorescence medium and the sample is acceptable, LCS based temperature sensing has the advantages of good spatial resolution to local temperature and convenient optical detection in the visible to near infrared wavelength range^40^,^41^,^42^,^43^,^44^,^45^,^46^,^47^.

On the other hand, ATX enables temperature measurement by sampling the near- or far-field radiation of the substrate without requiring any physical contact. Such non-contact temperature sensing is important for a wide range of applications from medical to industrial domains. Current techniques often employ semiconductor based infrared photon detectors or bolometer based detectors^54^,^55^. The limited detection range of various semiconductor materials and the slow response of bolometers restricts the application of these techniques^54^,^55^. Temperature sensing using ATX allows the use of visible to near infrared photo detectors to detect the upconverted fluorescence which are fast and widely available. Thus, ATX may enable temperature sensing with high spatial accuracy when combined with existing near-field scanning techniques^56^,^57^ by upconverting thermal radiation to near infrared or visible wavelengths for detection without requiring any physical contact with the sample.

## Summary

In summary, this work outlines the generalized theory of ATX and demonstrates a mathematical equivalence between LCS and ATX. With this equivalence, we compare the ideal efficiencies and up-converted power achieved for LCS with ATX. We find ATX potentially advantageous at higher temperatures for which the energy gap *δE* ~ *k*_*b*_*T*. The generalized model for ATX presented here will thus advance the understanding and application of utilizing active processes to manipulate near-field thermal radiation for thermal management.

## Additional Information

**How to cite this article**: Ding, D. *et al.* Active Thermal Extraction and Temperature Sensing of Near-field Thermal Radiation. *Sci. Rep.* **6**, 32744; doi: 10.1038/srep32744 (2016).

## References

[b1] Jenness J. Radiative cooling of satellite-borne electronic components. Proceedings of the IRE 48, 641–643 (1960).

[b2] Malkevich M. S., Pokras V. M. & Yurkova L. I. Measurements of radiation balance on the satellite Explorer VII. Planetary and Space Science 11, 839–865 (1963).

[b3] Granqvist C. G. Radiative heating and cooling with spectrally selective surfaces. Applied Optics 20, 2606 (1981). 20333006 10.1364/AO.20.002606

[b4] Hogan N. J. *et al.* Nanoparticles heat through light localization. Nano Lett. 14, 4640–4645 (2014). 24960442 10.1021/nl5016975

[b5] Ghasemi H. *et al.* Solar steam generation by heat localization. Nat. Commun. 5 (2014). 10.1038/ncomms5449 25043613

[b6] Zhu L., Raman A., Wang K. X., Anoma M. A. & Fan S. Radiative cooling of solar cells. Optica 1, 32–38 (2014).

[b7] Raman A. P., Anoma M. A., Zhu L., Rephaeli E. & Fan S. Passive radiative cooling below ambient air temperature under direct sunlight. Nature 515, 540–544 (2014). 25428501 10.1038/nature13883

[b8] Lenert A. *et al.* A nanophotonic solar thermophotovoltaic device. Nat. Nano. 9, 126–130 (2014). 10.1038/nnano.2013.286 24441985

[b9] Shen S., Narayanaswamy A. & Chen G. Surface phonon polaritons mediated energy transfer between nanoscale gaps. Nano Lett. 9, 2909–2913 (2009). 19719110 10.1021/nl901208v

[b10] Rousseau E. *et al.* Radiative heat transfer at the nanoscale. Nat. Photon. 3, 514–517 (2009).

[b11] Kim K. *et al.* Radiative heat transfer in the extreme near field. Nature 528, 387–391 (2015). 26641312 10.1038/nature16070

[b12] St-Gelais R., Zhu L., Fan S. & Lipson M. Near-field radiative heat transfer between parallel structures in the deep subwavelength regime. Nat. Nano 11, 515–519 (2016). 10.1038/nnano.2016.20 26950243

[b13] Shi J., Liu B., Li P., Ng L. Y. & Shen S. Near-field energy extraction with hyperbolic metamaterials. Nano Lett. 15, 1217–1221 (2015). 25622221 10.1021/nl504332t

[b14] Guha B., Otey C., Poitras C. B., Fan S. & Lipson M. Near-field radiative cooling of nanostructures. Nano Lett. 12, 4546–4550 (2012). 22891815 10.1021/nl301708e

[b15] Song B. *et al.* Enhancement of near-field radiative heat transfer using polar dielectric thin films. Nat. Nano. 10, 253–258 (2015). 10.1038/nnano.2015.6 25705866

[b16] Chen K., Santhanam P., Sandhu S., Zhu L. & Fan S. Heat-flux control and solid-state cooling by regulating chemical potential of photons in near-field electromagnetic heat transfer. Phys. Rev. B 91, 134301 (2015).

[b17] Pringsheim P. Zwei Bemerkungen über den unterschied von lumineszenz- und temperaturstrahlung. Z. Physik 57, 739–746 (1929).

[b18] Ding D., Kim T. & Minnich A. J. Active thermal extraction of near-field thermal radiation. Phys. Rev. B 93, 081402 (2016). 10.1038/srep32744 PMC5011705 27595609

[b19] Epstein R. I., Buchwald M. I., Edwards B. C., Gosnell T. R. & Mungan C. E. Observation of laser-induced fluorescent cooling of a solid. Nature 377, 500–503 (1995).

[b20] Mungan C. E., Buchwald M. I., Edwards B. C., Epstein R. I. & Gosnell T. R. Laser cooling of a solid by 16 k starting from room temperature. Phys. Rev. Lett. 78, 1030–1033 (1997).

[b21] Luo X., Eisaman M. D. & Gosnell T. R. Laser cooling of a solid by 21 K starting from room temperature. Optics Letters 23, 639 (1998). 18084602 10.1364/ol.23.000639

[b22] Gosnell T. R. Laser cooling of a solid by 65 K starting from room temperature. Optics Letters 24, 1041–1043 (1999). 18073934 10.1364/ol.24.001041

[b23] Hoyt C. W., Sheik-Bahae M., Epstein R. I., Edwards B. C. & Anderson J. E. Observation of anti-stokes fluorescence cooling in thulium-doped glass. Phys. Rev. Lett. 85, 3600–3603 (2000). 11030960 10.1103/PhysRevLett.85.3600

[b24] Fernàndez J., Mendioroz A., Garcìa A. J., Balda R. & Adam J. L. Anti-Stokes laser-induced internal cooling of TM^3+^ -doped glasses. Phys. Rev. B 62, 3213–3217 (2000).

[b25] Epstein R. I., Brown J. J., Edwards B. C. & Gibbs A. Measurements of optical refrigeration in ytterbium-doped crystals. Journal of Applied Physics 90, 4815–4819 (2001).

[b26] Rayner A., Friese M. E. J., Truscott A. G., Heckenberg N. R. & Rubinsztein-dunlop H. Laser cooling of a solid from ambient temperature. Journal of Modern Optics 48, 103–114 (2001).

[b27] Mendioroz A. *et al.* Anti-Stokes laser cooling in Yb^3+^-doped KPb_2_Cl_5_ crystal. Optics Letters 27, 1525 (2002). 18026494 10.1364/ol.27.001525

[b28] Heeg B. *et al.* Experimental demonstration of intracavity solid-state laser cooling of Yb^3+^:ZBLAN glass. Phys. Rev. A 70, 021401 (2004).

[b29] Thiede J., Distel J., Greenfield S. R. & Epstein R. I. Cooling to 208 K by optical refrigeration. Applied Physics Letters 86, 154107 (2005).

[b30] Bigotta S. *et al.* Laser cooling of Yb^3+^-doped BaY_2_F_8_ single crystal. Optical Materials 28, 1321–1324 (2006).

[b31] Bigotta S. *et al.* Spectroscopic and laser cooling results on Yb^3+^-doped BaY_2_F_8_ single crystal. Journal of Applied Physics 100, 013109 (2006).

[b32] Sheik-Bahae M. & Epstein R. I. Optical refrigeration. Nat. Photon. 1, 693–699 (2007).

[b33] Patterson W. *et al.* Anti-Stokes luminescence cooling of Tm^3+^ doped BaY_2_F_8_. Optics Express 16, 1704 (2008). 18542249 10.1364/oe.16.001704

[b34] Sheik-Bahae M. & Epstein R. Laser cooling of solids. Laser & Photon. Rev. 3, 67–84(2009).

[b35] Seletskiy D. V. *et al.* Laser cooling of solids to cryogenic temperatures. Nat. Photon. 4, 161–164 (2010).

[b36] Nemova G. & Kashyap R. Laser cooling of solids. Reports on Progress in Physics 73, 086501 (2010).

[b37] Roder P. B., Smith B. E., Zhou X., Crane M. J. & Pauzauskie P. J. Laser refrigeration of hydrothermal nanocrystals in physiological media. 112(49), 15024–15029 (2015). 10.1073/pnas.1510418112 PMC4679026 26589813

[b38] Zhang J., Li D., Chen R. & Xiong Q. Laser cooling of a semiconductor by 40 kelvin. Nature 493, 504–508 (2013). 23344360 10.1038/nature11721

[b39] Ha S.-T., Shen C., Zhang J. & Xiong Q. Laser cooling of organic–inorganic lead halide perovskites. Nat. Photon. 10, 115–121 (2016).

[b40] Saϊdi E. *et al.* Scanning thermal imaging by near-field fluorescence spectroscopy. Nanotechnology 20, 115703 (2009). 19420451 10.1088/0957-4484/20/11/115703

[b41] Vetrone F. *et al.* Temperature sensing using fluorescent nanothermometers. ACS Nano 4, 3254–3258 (2010). 20441184 10.1021/nn100244a

[b42] Carlson M. T., Khan A. & Richardson H. H. Local temperature determination of optically excited nanoparticles and nanodots. Nano Lett. 11, 1061–1069 (2011). 21306114 10.1021/nl103938u

[b43] Fischer L. H., Harms G. S. & Wolfbeis O. S. Upconverting nanoparticles for nanoscale thermometry. Angew. Chem. Int. Ed. 50, 4546–4551 (2011). 10.1002/anie.201006835 21495125

[b44] Sedlmeier A., Achatz D. E., Fischer L. H., Gorris H. H. & Wolfbeis O. S. Photon upconverting nanoparticles for luminescent sensing of temperature. Nanoscale 4, 7090–7096 (2012). 23070055 10.1039/c2nr32314a

[b45] Dong B. *et al.* Temperature sensing and *in vivo* imaging by molybdenum sensitized visible upconversion luminescence of rare-earth oxides. Adv. Mater. 24, 1987–1993 (2012). 22422477 10.1002/adma.201200431

[b46] Wang X., Zheng J., Xuan Y. & Yan X. Optical temperature sensing of NaYbF_4_: Tm^3+^@SiO2 core-shell micro-particles induced by infrared excitation. Opt Express 21, 21596–21606 (2013). 24104034 10.1364/OE.21.021596

[b47] Dong J. & Zink J. I. Taking the temperature of the interiors of magnetically heated nanoparticles. ACS Nano 8(5), 5199–5207 (2014). 24779552 10.1021/nn501250e PMC4046777

[b48] Mungan C. E. & Gosnell T. R. Laser cooling of solids. Advances in Atomic, Molecular, and Optical Physics 40, 161–228 (1999).

[b49] Ruan X. L. & Kaviany M. Advances in laser cooling of solids. Journal of Heat Transfer 129, 3 (2007).

[b50] Einstein A. Zur quantentheorie der strahlung. Physikalische Zeitschrift 18, 121–128 (1917).

[b51] Riseberg L. A. & Moos H. W. Multiphonon orbit-lattice relaxation of excited states of rare-earth ions in crystals. Phys. Rev. 174, 429–438 (1968).

[b52] Dijk J. M. F. v. & Schuurmans M. F. H. On the nonradiative and radiative decay rates and a modified exponential energy gap law for 4f–4f transitions in rare earth ions. The Journal of Chemical Physics 78, 5317–5323 (1983).

[b53] Hehlen M. P. Novel materials for laser refrigeration. Proc. of SPIE 7228, 72280E–1–72280E–8 (2009).

[b54] Childs P. R. N., Greenwood J. R. & Long C. A. Review of temperature measurement. Review of Scientific Instruments 71, 2959–2978 (2000).

[b55] Griffith B., Türler D., Goudey H. & Hornak J. P. Infrared thermographic systems. The Encyclopedia of Imaging Science and Technology (2001).

[b56] De Wilde Y. *et al.* Thermal radiation scanning tunnelling microscopy. Nature 444, 740–743 (2006). 17151664 10.1038/nature05265

[b57] Jones A. C. & Raschke M. B. Thermal infrared near-field spectroscopy. Nano Lett. 12, 1475–1481 (2012). 22280474 10.1021/nl204201g

